# Gastrocolic Fistula Due to Staple Line Leak Following Metabolic Bariatric Surgery: A Systematic Review

**DOI:** 10.1007/s11695-025-07844-2

**Published:** 2025-04-17

**Authors:** Mohamed Hany, Anwar Ashraf Abouelnasr, Muhammad Gaballah, Ahmed Ragab, Dina Mohamed Hafez, Bart Torensma

**Affiliations:** 1 Alexandria University, Medical Research Institute, Alexandria, Egypt; 2 Erasmus MC, Department of Epidemiology, Rotterdam, the Netherlands

**Keywords:** Gastric colic fistula, Staple line leak, Sleeve gastrectomy, One Anastomosis Gastric Bypass, Roux-en-Y gastric by pass

## Abstract

Gastrocolic fistula (GCF) is a rare but serious complication following metabolic bariatric surgery (MBS), primarily caused by leaks from the staple line or anastomotic sites. This systematic review aims to comprehensively analyze the diagnosis and management strategies for GCF following MBS. The Cochrane Central Register of Controlled Trials (CENTRAL), PubMed, Scopus, Web of Science, and EMBASE were searched until March 2024. Studies were independently screened and extracted using the PRISMA checklist. This systematic review included 16 studies (13 case reports and three retrospective cohort studies). GCF was most prevalent in laparoscopic sleeve gastrectomy (LSG), accounting for 81.2% of the studies. The transverse colon was the most common site of occurrence. Exploratory laparoscopy was utilized as the final treatment or rescue surgery in 85.7% of GCF case report studies, with all patients successfully healed following these interventions. The time from diagnosis to closure of the GCF varied significantly: in acute studies, closure occurred between 14 and 61 days, while in chronic studies, it ranged from 1 to 12 months. This systematic review concludes that surgical intervention should be recommended for managing GCF following specific staple line leaks in LSG. Most GCF studies occurred more than 6 months post-surgery, indicating a late complication. Computed tomography (CT) was the most frequently used diagnostic tool. Although over-the-scope clips (OTSC) and stents were employed, their efficacy as primary treatments remain questionable due to limited and inconsistent study outcomes.

**Supplementary Information:**

The online version contains supplementary material available at 10.1007/s11695-025-07844-2.

## Introduction

Obesity is a leading cause of morbidity and mortality, necessitating effective weight-loss interventions. Laparoscopic sleeve gastrectomy (LSG), Roux-en-Y gastric bypass (RYGB), and one-anastomosis gastric bypass (OAGB) are among the most performed metabolic bariatric surgeries (MBS) worldwide, offering significant weight reduction and improvement in associated medical problems [1].

Gastrocolic fistula (GCF) is a rare but serious complication of these procedures, primarily caused by leaks from the staple line or anastomotic sites. The etiology of GCF involves forming an abnormal connection between the stomach and the colon, often resulting from persistent collections below the diaphragm due to staple line leaks [2, 3].

Gastric leak rates from the sleeve staple line range from 0.1 to 7% for primary sleeve gastrectomies and 16 to 20% for re-operative surgeries following prior gastric procedures. Endoscopic intervention may be viable when fistulization after sleeve gastrectomy is identified early. However, as suggested by current studies, surgical re-intervention is almost invariably required in chronic fistula cases [4, 5].

The treatment of complex fistulas, such as GCF, is debated, and a multidisciplinary approach involving endoscopists, radiologists, and surgeons is mandatory. Early diagnosis and prompt management are crucial to improving patient outcomes and preventing severe complications [6].

Despite advancements in MBS techniques, managing GCF remains challenging. The condition’s rarity leads to limited experience and inconsistent management protocols among surgeons. Furthermore, the variability in patient presentations and many fistula studies complicate treatment decisions. Identifying leaks early is difficult due to the subtlety of symptoms and the delayed onset of complications, which can range from days to years post-surgery. This delay often results in patients presenting with well-established fistulas that are more resistant to less invasive treatments.

Radiological assessments, mainly CT scans, are vital in diagnosing GCF. However, they may occasionally fail to detect early or small leaks. Endoscopic techniques can be beneficial but often face limitations in chronic cases due to fibrosis and tissue remodeling around the fistula site, which can hinder their effectiveness [7].

Surgical re-intervention, usually necessary in these situations, carries its own risks and challenges, including the need for extensive adhesiolysis and the potential for further complications.

This systematic review aims to comprehensively analyze the diagnosis and management strategies for gastrocolic fistula following MBS.

## Methods

This systematic review (SR) was conducted according to the PRISMA Reporting Items for Systematic Reviews and Meta-Analyses (PRISMA) guidelines by Moher et al. [8] (Checklist appendix 1). All relevant and present studies of Gastrocolic Fistula after MBS studies were collected for this SR and were registered at Prospero [BLINDED].

### Study Aim

This study aims to systematically explore and map the existing literature on gastrocolic fistula following MBS on management and treatment outcomes.

### Inclusion and Exclusion Criteria

Inclusion Criteria: The review included studies involving patients aged 18 years or older with prior MBS treatment who suffer from complications of Gastrocolic Fistula (GCF) due to the staple line leak.

Exclusion criteria: Gastrocolic fistula due to marginal ulcers.

Objectives:

- Evaluate the clinical presentation of gastrocolic fistula in post-MBS
- Assess the diagnostic methods used to identify gastrocolic fistula in these patients
- Review the surgical and non-surgical management strategies for gastrocolic fistula

### Study Selection

The Cochrane Central Register of Controlled Trials (CENTRAL), PubMed, Scopus, Web of Science, and EMBASE were searched until March 2024. We used the following terms and their synonyms, which were truncated where necessary: “gastrocolic fistula “[MeSH Terms] OR “gastrocolic fistula” OR “gastro-colic fistula”) AND (“sleeve gastrectomy”[MeSH Terms] OR “sleeve gastrectomy” OR “bariatric surgery” OR “Roux-en-Y gastric bypass” OR “RYGB” OR “One Anastomosis Gastric Bypass” OR “OAGB” OR “Single Anastomosis Duodeno-Ileal bypass with Sleeve gastrectomy” OR “SADI-S”) AND (treatment OR management OR therapy OR outcome OR prevalence). Grey literature was also searched with a reference crosscheck to detect eligible articles not identified in the previous searches or SR. This search was conducted without restrictions on the language.

### Type of Studies

#### Included

Prospective and retrospective cohort studies and case reports were included.

### Data Extraction

Three reviewers (AAA, MG, AR) independently screened the titles and abstracts based on the inclusion criteria. Then, the same reviewers independently reviewed the remaining full-text reports for eligibility. In all stages, disagreements were resolved by discussion or consulting a fourth independent reviewer (BT).

### Assessment of Risk of Bias

Three reviewers (AAA, MG, AR) independently assessed the risk of bias for the methodological quality of each included study using the Critical Appraisal Skills Programme (CASP) checklist [9], while case reports were assessed using the “Case reports JBI critical appraisal checklist.” Eight items were evaluated for Yes, NO, Unclear, or Unapplicable, and the final decision was whether to include or exclude or seek further information. In all stages, disagreements were resolved by discussion or consulting an independent reviewer (BT).

### Statistical Analysis

All the studies were screened in Rayyan (Rayyan ai 2024; 10.1186/s13643-016-0384-4). Extraction was done in Jotform Inc. (4 Embarcadero Center, Suite 780, San Francisco, CA 94111) and analyzed in R studio with R markdown data.table, knitr, stringer, skimmer, and ggplot2 (Version 2023.06.0 + 421). Categorical variables were expressed as n (%). Continuous normally distributed variables were represented with their means and standard deviations, while non-normally distributed variables were described with their medians and min–max ranges. If appropriate, a meta-analysis was conducted. Heterogeneity was evaluated using the tau-square (*I*^2^) statistic. I^2^ of 0–40% was considered low heterogeneity, 30–50% moderate heterogeneity, 50–75% substantial heterogeneity, and 75–100% high heterogeneity, respectively. When heterogeneity was greater than 60% or outcomes were not displayed, a meta-analysis was not used, and the outcomes of the articles were described in the SR.

## Results

### Literature Search

**Fig. 1:**
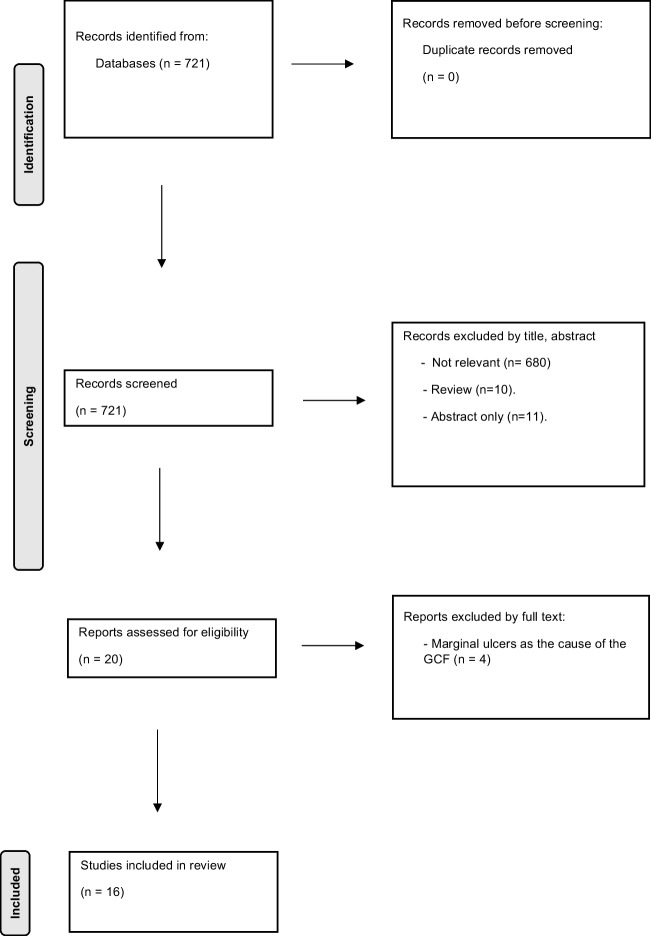
PRISMA 2020 flow diagram for systematic reviews

In total, 721 references were imported for screening, and zero duplicates were removed. Seven hundred two were excluded after screening their titles and abstracts. After evaluation, 20 studies were found; 4 studies were excluded due to marginal ulcers as the cause of the GCF. 16 full-text articles were included (13 case reports [10–22] and three retrospective cohorts [23–25] (Fig. 1).

### Characteristics of the Included Studies

From 2009 to 2021, 14 patients from 9 countries were described in case reports. The median age (range) was 45 years (27–59), and 10 (76.9%) were females. The primary surgeries performed were 11 sleeve gastrectomies (LSG) (84.6%), one conversion from gastric band to LSG (7.7%), and one biliopancreatic diversion with duodenal switch (7.7%). The location of the colic fistula varied between the studies, as shown in Table 1.

**Table 1:** Baseline case report studies characteristics

Author	Year	Study design	Country	*N* = GCF	Primary or revision	First type of surgery	Age (in years)	Gender (female)	Location of colic fistula
Mancini et al	2021	Case Report	France	1	Primary	LSG	42	0 (0%)	Between the esophagogastric junction and the left colon
Badaoui et al	2021	Case Report	USA	1	Primary	LSG	57	1 (100%)	Transverse colon
Ezzy et al	2021	Case Report	Germany	1	Primary	LSG	41	1 (100%)	Between the stapled line and the transverse colon
DeLong et al	2020	Case Report	USA	2	Revision	LSG	First patient 48 Second patient 46	2 (100%)	Within the colon
Mohammed et al	2020	Case Report	Iraq	1	Primary	LSG	48	1 (100%)	Between the remnant parts of the gastric fundus, the transverse colon and left lower lobe of the lung
Dugan et al	2020	Case Report	USA	1	Primary	LSG	37	0 (0%)	Between the distal gastric staple line and the transverse colon
Gari et al	2019	Case Report	Saudi Arabia	1	Revision	LSG	32	0 (0%)	Tubular structure connecting the upper part of the stomach and the colon
Parmar et al	2019	Case Report	UK	1	Primary	LSG	31	1 (100%)	LSG and splenic flexure
Ball et al	2018	Case Report	UK	1	Revision	LSG after band	59	1 (100%)	5 cm below gastro-oesophageal junction
El Sayegh et al	2018	Case Report	Lebanon	1	Primary	LSG	28	0 (0%)	Gastroesophageal junction
Garofalo et al	2016	Case Report	Canada	1	Primary	LSG	47	1 (100%)	In the proximal antrum that was communicating with the transverse colon
Bhasker et al	2014	Case Report	India	1	Primary	LSG	27	1 (100%)	Gastroesophageal junction and splenic flexure of the colon
Trelles et al	2009	Case Report	USA	1	Revision	biliopancreatic diversion with duodenal switch	54	1 (100%)	The UGI series showed contrast in the gastric sleeve communicating with the colon

*GJ*, gastro jejuno; *NA*, not applicable/not reported; *GJA*, gastrojejunal anastomosis; *LSG*, sleeve gastrectomy

Regarding the cohort studies, 87 patients were included from 2 countries, of which only 12 had gastrocolic fistula. The median age (range) was 43 years (20–65), the proportion of females ranged from 39 to 75%. LSG was performed in two studies (66.6%) and Roux-en-Y gastric bypass (RYGB) in one study (33.3%). The location of colic fistula was the colon in one study (33.3%), adherent to the pancreas in one study, not specified in the third study (33.3%). No mortalities were described (Table 2).

**Table 2:** Baseline cohort studies characteristics

Author	Year	Study design	Country	Total sample	*N* = GCF	Primary or revision	First type of surgery	Age (in years)	Gender (female)	Location of colic fistula
D’Alessandro et al	2021	Retrospective cohort	France	40	10	Primary	LSG	For ten males, mean age was 37.5 ± 9 years old and mean BMI before LSG was 40.4 ± 6.1	30 (75%)*	Colon
Donatelli et al	2016	Retrospective cohort	France	33	1	Primary	RYGB	43 (20–65)	13/33 (39%)*	NA
Tan et al	2009	Retrospective cohort study	Australia	14	1	Revision	LSG	44 (26–64)*	10 (71%)*	Adherent to the pancreas

^*^It is unknown what the specific age and type of gender of the GCF patients are. This was not specified in the studies

*GJ*, gastro jejuno; *NA*, not applicable/not reported; *GJA*, gastrojejunal anastomosis; *LSG*, sleeve gastrectomy; *RYGB*, Roux-en-Y gastric bypass

### Signs and Symptoms

In total, 13 signs and symptoms were identified in the patients with GCF. All the case report studies were described as chronic diseases. Fever was present in 4 patients (28.6%), abdominal pain in 6 (42.9%), vomiting in 4 (28.6%), diarrhea in 5 (35.75), and dark maroon stool in 7.1% (Table 3).

**Table 3:** Signs and symptoms of patients in case reports

Author	Chronic case	fever	Abdominal pain	Vomiting	Chest pain	Colic	Cuffing	Complaints in the hospital	diarrhoea	Sepsis	Hematemesis	Nausea	Dark maroon stool	Left Shoulder pain
Mancini et al	**Y**	N	**Y**	N	N	N	N	N	N	N	N	N	N	N
Badaoui et al	**Y**	N	N	**Y**	N	N	N	N	**Y**	N	N	N	N	N
Ezzy et al	**Y**	N	**Y**	N	N	N	N	N	**Y**	N	N	N	N	**Y**
DeLong et al. 1/2	**Y**	N	N	N	N	N	N	**Y**	N	N	N	N	N	N
2/2	**NA**	**NA**	**NA**	**NA**	**NA**	**NA**	**NA**	**NA**	**NA**	**NA**	**NA**	**NA**	**NA**	**NA**
Mohammed et al	**Y**	**Y**	N	N	**Y**	N	**Y**	N	N	N	N	N	N	N
Dugan et al	**Y**	Not mentioned												
Gari et al	**Y**	N	**Y**	**Y**	N	N	N	N	N	**Y**	N	N	N	N
Parmar et al	**Y**	N	**Y**	N	**Y**	N	N	N	N	N	N	N	N	N
Ball et al	**Y**	N	N	N	N	N	N	**Y**	N	**Y**	**Y**	N	**Y**	N
El Sayegh et al	**Y**	**Y**	N	**Y**	**Y**	N	N	N	**Y**	N	N	N	N	**Y**
Garofalo et al	**Y**	N	N	N	N	N	N	N	**Y**	N	N	N	N	N
Bhasker et al	**Y**	**Y**	**Y**	N	N	N	N	N	**Y**	N	N	**Y**	N	**Y**
Trelles et al	**Y**	**Y**	**Y**	**Y**	N	N	N	N	N	N	N	**Y**	N	N

*Y* Yes, *N* No

Regarding the cohort studies, fever was present in 33.3%, abdominal pain in 33.3%, cuffing in 33.3%, diarrhea in 33.3%, and sepsis in 66.6% (Table 4).

**Table 4:** Signs and symptoms of patients in cohort studies

Author	Chronic case	fever	Abdominal pain	vomiting	Chest pain	Colic	Cuffing	Complaints in the hospital	diarrhea	Sepsis	Hematemesis	Nausea	Dark maroon stool	Left shoulder pain
D’Alessandro et al	**Y**	**Y**	**Y**	N	N	N	**Y**	N	**Y**	**Y**	N	N	N	N
Donatelli et al	N	N	N	N	N	N	N	N	N	**Y**	N	N	N	N
Tan et al	**Y**	N	N	N	N	N	N	**Y**	N	N	N	N	N	N

*Y* Yes, *N* No

### Type of Intervention(s)

In 9 out of 13 studies (69.2%), exploratory laparoscopy was performed as the final treatment or as rescue surgery for treating GCF. Seven out of 13 studies (53.8%) conducted another intervention first (stent, dilation, over-the-scope clip) before converting to exploratory laparoscopy. Five studies (38.5%) immediately performed exploratory laparoscopy, resulting in diverse surgical interventions. All patients successfully healed following these interventions (Table 5).

**Table 5:** Summary of type of intervention(s) and fistula treatment(s) in the case reports

Author	Time until complication from index surgery	Therapy duration until leak free	Type of intervention(s)	Fistula treatment(s)
Mancini et al	24 months	10 days	salvage robotic Roux-en-Y fistulojejunostomy The surgery was performed using the da Vinci® X system The intervention began with complete dissection of the sleeved stomach	The surgical management involved dissection of the right side of the sleeved stomach to the left crus of the diaphragm for isolation of the gastrocolic fistula. The colic defect was repaired using 00 simple interrupted sutures. The jejunum was transected 70 cm distal to the duodenojejunal junction, and the distal segment was anastomosed to the gastric fistula with 000 running sutures. Subsequently, the proximal jejunum was anastomosed to the distal jejunum 80 cm distal to the fistulojejunal anastomosis using 000 running sutures
Badaoui et al	108 months	3 months	laparoscopic conversion to Roux-enY-gastric bypass (RYGB) Additionally, due to the involvement of the splenic hilum with the inflammatory process, the patient was consented and counseled on a potential laparoscopic splenectomy	A laparoscopic approach was undertaken, involving the lysis of plastering adhesions, isolation and transection of the fistula tract, followed by the construction of a laparoscopic Roux-en-Y gastric bypass. The procedure necessitated a splenectomy as an inherent part of the surgical intervention
Ezzy et al	10 months	1 month	1. Endoscopy 2. CT scan 3. Exploratory laparoscopy + intraoperative gastroscopy	Small fistula tracts were identified between the proximal staple line, the left diaphragm, and the transverse colon, forming a gastrocolo-diaphragmatic fistula. Both fistulae were surgically resected, and the defect in the transverse colon was repaired using a two-layer suture technique. Peritoneal lavage was performed, the abscess cavity was drained, and a drain was placed to facilitate postoperative management. To enhance healing, an Endo-Sponge (polyurethane sponge) was endoscopically placed at the fistula opening to reduce intragastric pressure and promote granulation. Continuous suction at 100 mmHg was applied via drainage tubes connected to the sponge, which was replaced every three days. Postoperatively, the patient was started on a regimen of antibiotics and parenteral nutrition to support recovery
DeLong et al	NA	NA	First patient: 1. Upper and lower endoscopy 2. colonoscope Second patient: 1. Upper and lower endoscopy 2. Fluoroscopy 3. ultra slim trans-nasal scope 4. paediatric colonoscope	First patient: 12/6 T type Ovesco clip was fired on the colonic side and a 12/6 T type Ovesco clip was placed on the gastroscope and positioned over the fistula
Mohammed et al	60 months	6 months	1. At 48 months: Endoscopy stent placement 2. At 60 months CT + endoscopy 3. Exploratory laparoscopy and thoracotomy	The fistulous tract was surgically resected, and the defects in the stomach and colonic walls were repaired using a two-layer closure technique with absorbable sutures
Dugan et al	beyond 12 weeks	4 months	1. Non-operative management failed 2. Exploratory laparoscopy	A near-total gastrectomy was performed, preserving a small gastric pouch. The colonic aspect of the fistula was oversewn, and reconstruction was completed using a Roux-en-Y technique
Gari et al	3 years	6 weeks	1. Image-guided percutaneous drainage failed due to close proximity of the transverse colon to the abscess cavity 2. Exploratory laparoscopy	The gastrocolic fistula was meticulously dissected and excised using a linear stapler. Six weeks later, an open esophagojejunostomy combined with a total gastrectomy was successfully performed
Parmar et al	6 months	12 months	1.Oesophagogastroduodenoscopy confirmed the findings of colic fistula 2. Exploratory laparoscopy	Laparoscopic fistulectomy was performed, including adhesiolysis and mobilization of the gastric sleeve and splenic flexure. The fistulous tract was isolated, divided, and excised along with a portion of the colonic lumen
Ball et al	22 weeks	2 months	1. After conversion from band to LSG, a gastric-colic fistula to the splenic flexure of the large bowel occurred 2. A covered stent was inserted to manage the fistula 3. Heavy rectal bleeding occurred two weeks later 4. Oesophagogastroduodenoscopy (OGD) was performed but did not demonstrate a bleeding site 5. CT scan confirmed a pseudoaneurysm 6. Selective embolization was performed to address the pseudoaneurysm of the splenic vessel	The pseudoaneurysm was cannulated and successfully embolized using multiple microcoils. A follow-up CT scan performed two weeks later confirmed resolution of the fistula and the associated collection. Subsequently, the covered stent was removed endoscopically, and a nasojejunal feeding tube was placed to facilitate enteral nutrition
El Sayegh et al	8 months	3.5 months	1. CT scan confrmed fistula 2. Exploratory laparoscopy	Laparoscopic esophagogastrostomy with a Roux-en-Y esophagojejunal reconstruction
Garofalo et al	48 months	9 months	upper endoscopy, colonoscopy, barium study, computed tomography of the abdomen and pelvis	Endoscopic treatment using an over-the-scope clip (Ovesco Endoscopy, Germany) was attempted but was unsuccessful due to the fibrotic nature of the fistula. Subsequently, laparoscopic resection of the gastrocolic fistula was performed, accompanied by omental interposition and perioperative endoscopy to ensure thorough management
Bhasker et al	8 weeks	20 days	1. Endoscopic dilatation of the fistulous tract 2. 18 months later exploratory laparoscopy	1.The procedure was performed using a Controlled Radial Expansion (CRE) balloon (Boston Scientific, Marlborough, USA), allowing for internal drainage of the abscess into the stomach 2. The colonic fistula was surgically resected, and the staple line was reinforced with oversewing
Trelles et al	12 months	7 days	1. Endoscopic treatment 2. after 3 weeks an exploratory laparoscopy	1. Upper endoscopy was used to identify the fistula, after which a guidewire was inserted, and a nitinol silicone-covered stent (20 mm × 100 mm) was deployed under fluoroscopic guidance 2. The fistula was subsequently repaired using a laparoscopic approach

*NA* Not Applicable/not reported, *GJ* Gastro Jejuno, *GJA* gastrojejunal anastomosis, *LSG* Sleeve Gastrectomy

In the retrospective cohort study by D’Alessandro et al., initially, an upper gastrointestinal endoscopy was performed. Subsequently, one or more double pigtail stents (Endoflex®, Cook Medical®) were introduced. After 4–6 weeks, a CT scan was conducted to confirm healing. If the fistula persisted after 1 year, endoscopic treatment was deemed inefficient, and surgical intervention was indicated. In studies where the double pigtail treatment failed after 12 months, exploratory laparoscopy was performed as rescue surgery in 52.5% of the patients.

In the retrospective cohort study by Donatelli et al., only one case (4.6%) in which a secondary complex fistula (gastro-gastric, gastrocolic) developed. This necessitated endoscopic necrosectomy, re-stenting with a pigtail stent, and the placement of a nasojejunal feeding tube for an additional 4 weeks before transitioning to a normal diet. The definitive removal of the pigtail happened after 99 days of treatment.

The retrospective cohort study by Tan et al. included 14 patients with diverse leaks. One of them was described as a GCF. The study employed a variety of interventions, including laparotomy, laparoscopy, endoscopic covered stenting, percutaneous radiologically guided drainage, jejunal enteric feeding, and total parenteral nutrition. Still, it is not clear what treatment the GCF got. It described the implementation of an algorithm-based multidisciplinary team approach for managing gastrocutaneous fistulas (GCF). However, the study had low power in determining the most effective decision-making strategy (Table 6).

**Table 6:** Summary of type of intervention(s) and fistula treatment(s) in the cohort studies

Author	Time until complication from index surgery	Therapy duration until leak free	Type of intervention(s)	Fistula treatment(s)
D’Alessandro et al	265.6 ± 521 days	16 months	1. Upper gastrointestinal endoscopy 2. One or more double pigtails (Endoflex®, Cook Medical®) were introduced 3. CT scan to confirm the healing after 4–6 weeks 4. persistence of fistula after 1-year, endoscopic treatment was considered inefficacy and surgical intervention was indicated 5. Exploratory laparoscopy	One or more double pigtail stents (EndoFLEX®, Cook Medical®) were inserted through the orifice of the fistulous tract to facilitate drainage and promote second-intention healing or to occlude the tract
Donatelli et al	10 (4–35) days	61 (28–99) days	1. Endoscopic stenting	In 4.6% of cases (one patient), a complex fistula (gastro-gastric and gastrocolic) developed. Management included endoscopic necrosectomy, placement of a pigtail stent, and insertion of a nasojejunal feeding tube for nutritional support over four weeks. The patient was transitioned to a normal diet before the definitive removal of the pigtail stent, resulting in a total treatment duration of 99 days
Tan et al	NA	NA	Unknow what treatment was used for the one GCF patient	GCF was disconnected

*NA* Not Applicable/not reported, *GJ* Gastro Jejuno, *GJA* gastrojejunal anastomosis, *LSG* Sleeve Gastrectomy

### Time of Onset and Until Leak Free

Mancini et al., Badaoui et al., Mohammed et al., Gari et al., and Garofalo et al. presented longer than 12 months after index surgery (24, 108, 60, 36, and 48 months) (10 days, 3 months, 6 months, 6 weeks, and 9 months until leak free). Ball et al., Bhasker et al., and Trelles et al. presented studies within 6 months from index surgery (22, 8 weeks, 12 months of onset) (2 months, 20 days, 7 days leak free) as shown in Table 5.

D’Alessandro et al. presented 265.6 ± 521 days of onset 16 months until leek free; Donatelli et al. presented acute studies within 10 (4–35) days and leak free after 61 (28–99) days after secondary complex GCF. Tan et al. did not provide a timeline for the onset of GCF (Table 6).

### Antibiotic Treatment

Regarding the case–control studies, six out of 13 studies presented antibiotic (AB) treatment (Badaoui et al., Mohammed et al., Ezzy et al., Gari et al., El Sayegh et al., and Trelles et al.). Only one study (El Sayegh et al.) provided detailed AB therapy (ceftriaxone, and levofloxacin/piperacillin/tazobactam and fluconazole/co-amoxiclav then piperacillin/tazobactam and fluconazole then co-amoxiclav). The other five studies did not describe it or were mentioned as a “broad spectrum.” None of the studies described the dosage or duration (Table 7).

**Table 7:** Antibiotic use, Clavien Dindo, and intensive care admission in the case reports

Author	AB treatment	Type of AB and days	Complications Clavien Dindo classification	ICU admittance		IF YES ICU duration
Mancini et al	N	N	CD3b	Y	NA	
Badaoui et al	Y	2 years	CD3b	N	N	
Ezzy et al	Y	NA	CD3b	N	N	
DeLong et al	N	N	CD3b	N	N	
Mohammed et al	Y	N	NA	Y	3 days	
Dugan et al	N	N	CD3b	N	N	
Gari et al	Y	empirical and antifungal	CD3b	Y	NA	
Parmar et al	N	N	CD3b	N	N	
Ball et al	N	N	CD3b	Y	NA	
El Sayegh et al	Y	ceftriaxone, and levofloxacin/piperacillin/tazobactam and fluconazole/co-amoxiclav then piperacillin/tazobactam and fluconazole then co-amoxiclav	CD3b	N	N	
Garofalo et al	N	N	CD3b	Y	NA	
Bhasker et al	N	N	CD3b	N	N	
Trelles et al	Y	First postoperative week the patient received medical treatment with appropriate antibiotics, One month later the patient required 4 days of hospitalization for intravenous antibiotics	CD3b	Y	4 days	

*GJ* Gastro Jejuno, *NA* Not Applicable/not reported, *GJA* gastrojejunal anastomosis, *LSG* Sleeve Gastrectomy *Y* Yes, *N* No

Regarding the cohort studies, one of the three presented AB treatment for 5 days (D’Alessandro et al.) (Table 8).

**Table 8:** Antibiotic use, Clavien Dindo, and intensive care admission in the cohort studies

Author	AB treatment	Type of AB and days	Complications Clavien Dindo classification	ICU admittance	IF YES ICU duration
D’Alessandro et al	Y	5 days	CD3b	Y	NA
Donatelli et al	N	N	CD3b	N	N
Tan et al	N	N	CD3b	Y	NA

*Y* Yes, *N* No

### Intensive Care Unit

Six cases out of 13 case reports were admitted to the intensive care unit, where Mohammed et al. and Trelles et al. described the duration as three and four days, respectively. The rest was unknown (Table 7).

In the cohort studies, two out of three mentioned that patients were admitted to the ICU (D’Alessandro et al., Tan et al.,), as shown in Table 8.

### Type of Stent, Drain, or Clip Use

In the case reports, three of 13 studies described stent use as a treatment option. Two studies (Parmar et al., and Ball et al.) described a covered stent, but the material was not defined. Trelles et al. used a partially covered, self-expanded metal esophageal stent, and a nitinol silicone-covered stent, 20 mm × 100 mm (Alimaxx-E Esophageal Stent System, Alveolus, Charlotte, NC).

In the cohort studies, two studies out of three described stent use as a treatment option. Tan et al. described a covered stent, but the material was not defined. Donatelli et al. used double pigtail stent ((3 to 10 cm long, 7–8.5–10 Fr diameter) (Visio® Gflex Europe, Nivelles-Belgium or Advanix® Boston Scientific, Massachusetts-Boston-USA) for secondary complex GCF.

## Risk of Bias Assessment of Cohort Studies

**Fig. 2:**
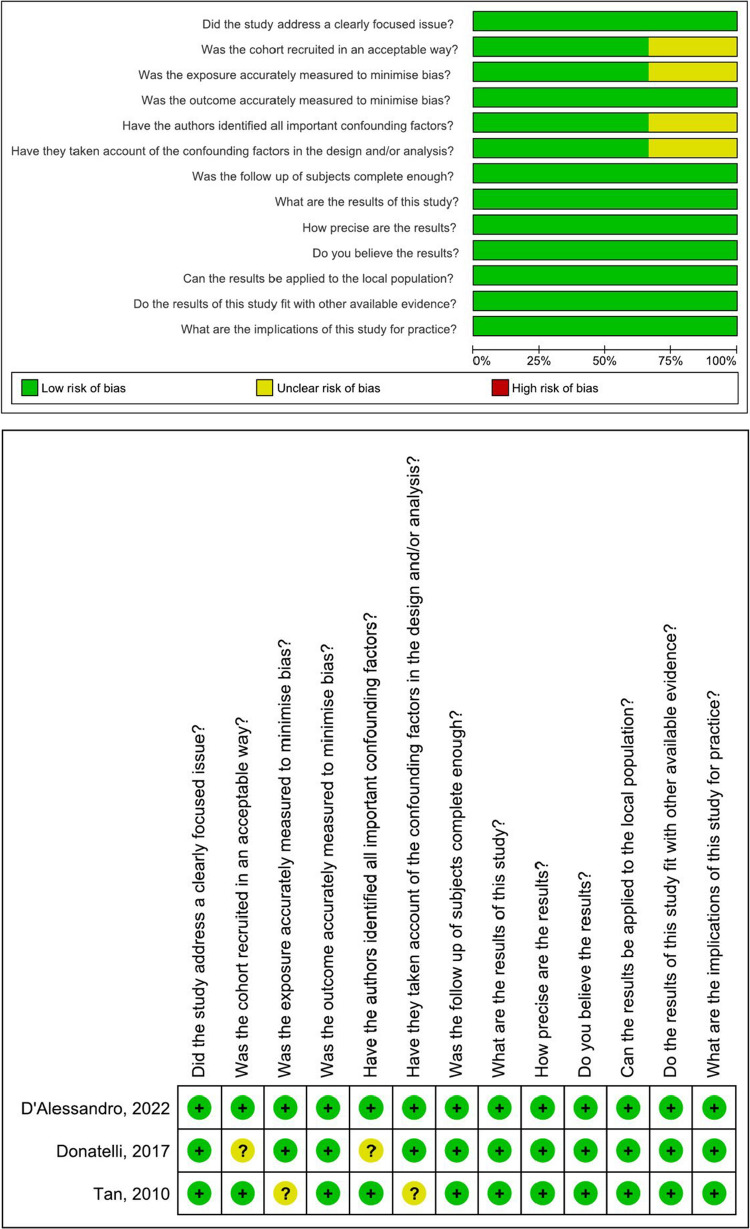
Risk of bias summary and graph of the cohort studies

The risk of bias assessment for the three cohort studies was performed using the CASP checklist. The studies exhibited a low to moderate risk of bias (Fig. 2).

## Quality Assessment of Case Reports

**Fig. 3:**
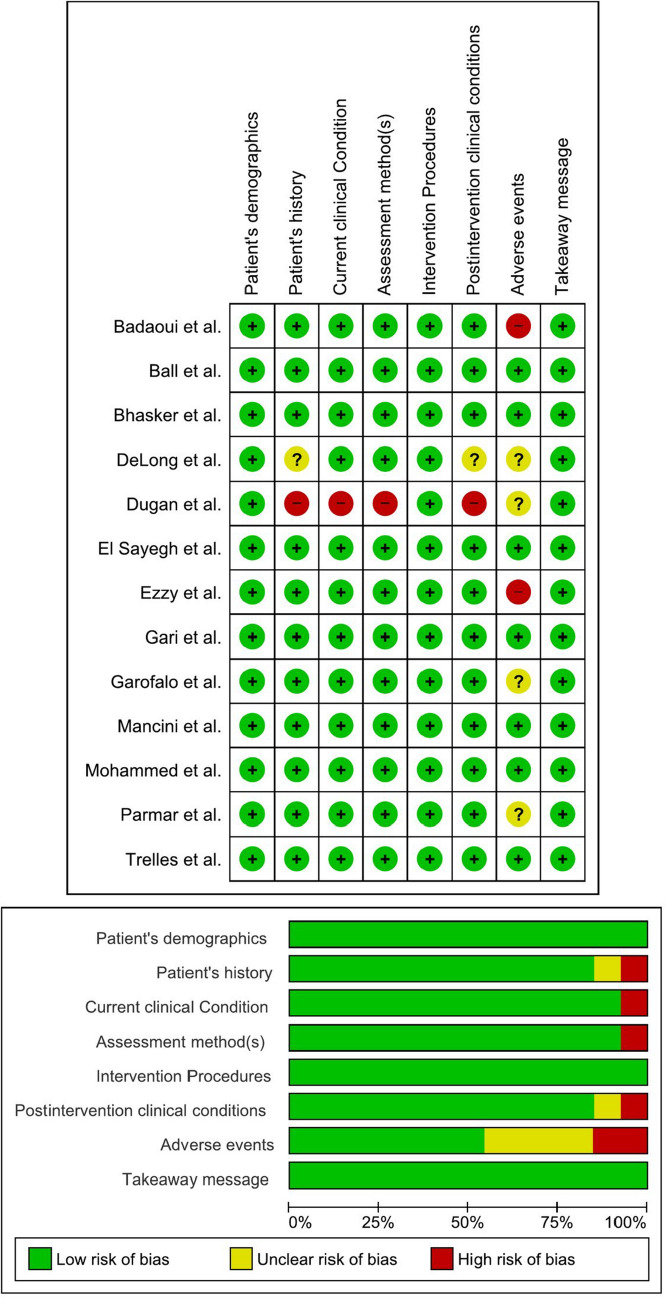
Risk of bias summary and graph of the case report studies

Most studies, including those by Mancini, Mohammed, Gari, and others, provide clear descriptions across all domains, reflecting thorough reporting. However, certain studies, like those by Dugan and DeLong, reveal gaps in specific areas, such as post-intervention conditions and identifying adverse events. Therefore, most case reports offer valuable lessons, though attention to detail in all aspects would enhance their utility for clinical practice (Table 9, Fig. 3).

**Table 9:** Case reports critical appraisal

Study	D1/Were patient’s demographic characteristics clearly described?	D2/Was the patient’s history clearly described and presented as a timeline?	D3/Was the current clinical condition of the patient on presentation clearly described?	D4/Were diagnostic tests or assessment methods and the results clearly described?	D5/Was the intervention(s) or treatment procedure(s) clearly described?	D6/Was the post-intervention clinical condition clearly described?	D7/Were adverse events (harms) or unanticipated events identified and described?	D8/Does the case report provide takeaway lessons?
Mancini et al	Yes	Yes	Yes	Yes	Yes	Yes	Yes	Yes
Badaoui et al	Yes	Yes	Yes	Yes	Yes	Yes	No	Yes
Ezzy et al	Yes	Yes	Yes	Yes	Yes	Yes	No	Yes
DeLong et al	Yes	Unclear	Yes	Yes	Yes	Unclear	Unclear	Yes
Mohammed et al	Yes	Yes	Yes	Yes	Yes	Yes	Yes	Yes
Dugan et al	Yes	No	No	No	Yes	No	Unclear	Yes
Gari et al	Yes	Yes	Yes	Yes	Yes	Yes	Yes	Yes
Parmar et al	Yes	Yes	Yes	Yes	Yes	Yes	Unclear	Yes
Ball et al	Yes	Yes	Yes	Yes	Yes	Yes	Yes	Yes
El Sayegh et al	Yes	Yes	Yes	Yes	Yes	Yes	Yes	Yes
Garofalo et al	Yes	Yes	Yes	Yes	Yes	Yes	Unclear	Yes
Bhasker et al	Yes	Yes	Yes	Yes	Yes	Yes	Yes	Yes
Trelles et al	Yes	Yes	Yes	Yes	Yes	Yes	Yes	Yes

## Discussion

This systematic review (SR) included 16 studies. GCF was most prevalent in LSG, accounting for 84.6% of the studies, one case after the gastric band was removed and converted to LSG, and one biliopancreatic diversion with duodenal switch. In the case reports, it was not diagnosed after RYGB and OAGB. In a cohort study, only one RYGB was described as a complication arising from a secondary complex GCF following stent placement for a primary cardia leak. The transverse colon was identified as the most common site of occurrence, and exploratory laparoscopy was utilized as the final treatment or rescue surgery.

One of the primary causes of GCF is the failure to maintain proper staple lines and anastomotic integrity during MBS. Early detection and management of leaks or infections are crucial in significantly reducing the risk of fistula formation. However, this systematic review revealed that early detection was infrequent, highlighting a critical area for improvement in clinical practice.

The time from the index surgery to the development of GCF varies widely. Acute studies (25.0%) can develop GCF as early as 10 days post-surgery (in one RYGB case of secondary complex GCF), while chronic studies can take up to 9 years, with 75% occurring more than 6 months post-surgery. The time from diagnosis to closure of the GCF also varies significantly. In acute studies, closure occurs between 14 and 61 days, while in chronic studies, it ranges from 1 to 12 months. Previous systematic reviews (SRs) conducted by Sakran et al. and Bawa et al. reported differing resolution times for fistula formation in the chest and gastrocutaneous fistulas, respectively. Bawa et al. documented a median time to closure for gastrocutaneous fistulas of 81 days. In contrast, Sakran et al. reported a wide range of closure times for chest fistulas, spanning from 2 days to 7 years, with 47.8% of cases remaining unresolved beyond six months [6, 26]. Thus, across all SRs, the resolution times for different types of fistulas were unpredictable, varying significantly between short and long periods.

The unpredictable evolution of leaks and fistulas makes management challenging. GCF, in particular, should be considered a late possible complication rather than an acute disease, given its potential to manifest long after the initial surgery.

Due to its anatomical position, leaks after LSG typically occur in the upper part of the staple line. The published incidence of leaks in LSG is between 0.1 and 7%, with a decrease in recent years, primarily due to experience and the standardization of the surgical technique [4, 5]. These leaks can result in the persistence of collections below the diaphragm, leading to pathological communication between the stomach and the transverse or left-angle colon, thereby causing GCF.

If these leaks are refractory or left untreated, they are prone to become persistent and evolve into chronic fistulas. This progression is often due to the increased intraluminal pressure within the stomach, a result of its tubular shape post-surgery, even in the absence of strictures at the gastric incisura [27].

It is suggested that proper surgical techniques are essential to prevent such complications, including high-quality staplers and thorough intraoperative leak testing. While our study did not have access to specific data regarding the types of staplers used in primary surgeries, we acknowledge that the quality of staplers can vary significantly between manufacturers, even though all must meet basic regulatory standards such as CE marks. This variability in quality could influence surgical outcomes, although this hypothesis was beyond the scope of our current study and requires further investigation. Nevertheless, ensuring reliable, high-quality staplers is critical in minimizing the risk of leaks. Additionally, gentle handling of tissues to reduce trauma, reinforcement of the staple line, and ensuring an adequate blood supply are crucial to maintaining anastomotic integrity and promoting healing during MBS. While these measures can significantly reduce the risk of complications, they cannot provide complete protection. The anatomical location of upper gastrointestinal tract leaks and their proximity to critical abdominal and thoracic structures accounts for the heightened risk of fistulization when these leaks are not promptly diagnosed and managed [15, 28].

Nevertheless, the relationship between the anatomical location of upper gastrointestinal tract leaks and the onset of GCF in this SR remains unclear. We can hypothesize that tissue healing was complete, as most studies occurred more than six months after the index surgery. Despite this, the formation of fistulas was consistently observed along the staple line to the transverse colon in 31.3% of the studies described in this SR. This pattern suggests that factors beyond initial tissue healing, such as the mechanical stress at the staple line or persistent subclinical inflammation, may play a role in the development of GCF, but it stays unclear. For other fistulas that develop after MBS, gastrocutaneous fistulas commonly form from the proximal third of the gastric remnant. This condition is associated with extended hospitalizations and significant morbidity and mortality, with an incidence reported to range between 1.7 and 4.0% [29, 30]. Gastric fistulas in the chest following LSG present unique challenges due to the distinct physiology of the sleeved stomach. The high-pressure system, resulting from an intact pylorus at the distal end and a lower esophageal sphincter at the proximal end, contributes to the increased risk of persistent leaks or fistulation into adjacent anatomical compartments. Additionally, several other factors can contribute to the formation of gastric fistulas, including iatrogenic injuries, improper vascularization, ischemia, hematoma formation, technical issues, and staple misfiring [6, 31, 32].

### Treatment Options

The management of GCFs typically necessitates a multimodal approach, incorporating both non-surgical and surgical interventions based on the severity and complexity of the condition. Non-surgical management is the preferred initial strategy for well-controlled leaks without hemodynamic instability. This approach includes drainage, broad-spectrum antibiotic therapy, and adequate nutritional support to control local sepsis and facilitate healing [33, 34]. This SR confirms the use of a nasojejunal tube for enteral nutrition. Nevertheless, this SR shows that exploratory laparoscopy was necessary in almost all studies (81.3%). And seven out of 16 studies (43.75%) conducted another intervention first (stent, dilation, over-the-scope clip) before converting to exploratory laparoscopy.

### Stent and Drain Placement

A systematic review by Hernández et al. reported an overall success rate of 85.89% (95% CI, 82.52–89.25%) for leak closure, with a median interval of 44 days between stent placement and removal. Stent migration occurred in 18.65% of cases (95% CI, 14.32–22.98%), while the proportion of re-operations was 13.54% (95% CI, 9.94–17.14%), typically involving rescue surgery for leaks that failed to resolve with stent management. The review highlighted that most leaks were at the angle of His In LSG and some RYGB procedures. The authors concluded that the endoscopic placement of self-expanding stents is an effective option for managing leaks after MBS in selected patients, demonstrating high efficacy and low associated mortality rates [35].

Another SR examined the effectiveness of Endoscopic Internal Drainage (EID) treatment in MBS. The overall success rate for EID treatment was 91.6% for leak closure, with a median treatment duration of 78.4 days (50.1–106.7 days). Complications associated with EID were reported in four studies and included stenoses (*n* = 8), perforation (*n* = 1), esophageal ulceration (*n* = 3), bleeding (*n* = 2), and splenic hematoma (*n* = 1). Across all studies, 232 patients were included, with 80% having undergone LSG and 20% having undergone RYGB [36]. Nevertheless, reducing stent migration and re-operation rates is a significant challenge in both SRs [35, 36].

This SR was shown in the study by Donatelli et al., where internal pigtail placement in RYGB was used and the one case of GCF. A complication led to the development of a complex GCF, which required endoscopic necrosectomy, pigtail resenting, and a nose-jejunal feeding tube for four more weeks before being put on a regular diet and definitive removal of the pigtail for an overall 99 days of treatment. The GCF healed but persisted in a sub-clinic GGF.

One other study by Trellis et al. used a nitinol silicone-covered stent, but this needed a laparoscopic repairment to treat and heal the GCF [24]. In the cohort study by D’Alessandro et al., 52.5% of cases experienced failure of one or more double pigtail catheter treatments after 12 months, necessitating laparoscopic intervention as rescue surgery in all such instances [23]. Although other SRs have demonstrated positive responses to stent and drain treatments for leaks and fistulas using various diagnostic methods, this SR can conclude, even with the limited amount of evidence and power of the studies and the late onset and challenging anatomical position of GCF, stent placement appears to be neither recommended nor sufficiently effective.

### Over-the-Scope Clip (OTSC)

The OTSC system is an endoscopic technique involving a clip to close the fistula opening. Studies have demonstrated its effectiveness in studies where conventional stenting fails or is not feasible.

An SR by Shoar et al. tested the OTSC in managing leaks and fistulas in patients who underwent LSG. The time between LSG and leak/fistula ranged from 1 to 803 days. Most of the leaks/fistulas were located at the proximal staple line, with sizes ranging from 3 to 20 mm. The time between leak diagnosis and OTSC clipping ranged from 0 to 271 days. Thirty-three out of 53 patients (63.5%) required one clip to close the lesion.

Regarding OTSC-related complications, a leak occurred in five patients (9.3%), and OTSC migration, stenosis, and tear occurred in one patient each (1.8%). Of the 73 patients with post-LSG leaks treated with OTSC, 63 had an overall successful closure (86.3%). The OTSC system is a promising endoscopic approach for managing post-LSG leaks in appropriately selected patients. Unfortunately, most studies are series with small sample sizes, short-term follow-up, and mixed data of concomitant procedures with OTSC. Further studies should distinguish the net efficacy of the OTSC system from other concomitant procedures in treating post-LSG leaks [37].

This SR included one study by Delong et al. that used OTSC. In this case, Ovesco clips were applied to both ends of the fistula tract, and post-procedure fluoroscopy did not demonstrate a leak after 62 days [13]. A study by Garofalo et al. also used OTSC but failed due to the fibrotic nature of the fistula. Eventually, laparoscopic resection of the gastrocolic fistula with omental interposition and perioperative endoscopy was necessary [20].

Given that the systematic review by Shoar et al. was unable to draw definitive conclusions about the effectiveness of OTSC and excluded GCF from its analysis, coupled with findings from this review indicating its use in only 2 out of 100 patients with a success rate of 50%, OTSC does not appear to be the first-line treatment choice. Its application for GCF remains highly debatable.

### Surgical Interventions

When endoscopic methods are unsuccessful or contraindicated, alternative treatments must be considered. Endoscopic procedures may be contraindicated in studies of severe hemodynamic instability, extensive tissue necrosis, severe infection or sepsis, anatomical abnormalities that prevent safe endoscopic access, or when there is a risk of perforation that cannot be managed endoscopically; surgical repair remains the definitive treatment for GCF.

Surgical options may include resection of the fistula tract, re-anastomosis, and staple line reinforcement. In severe studies, a more extensive procedure, such as partial gastrectomy or colectomy, may be necessary. Surgical outcomes can be favorable, but the approach is associated with higher morbidity and longer recovery times. Predictors of success in surgical treatment include the patient’s overall health status, absence of severe associated medical problems, the timing of the intervention, and the surgeon’s expertise. Early identification and management of complications, as well as implementing a multidisciplinary care approach, also contribute to improved outcomes.

In terms of results, successful surgical interventions often lead to the resolution of the underlying issue, but extended hospital stays and increased morbidity rates typically accompany them. The duration of hospital stay can vary significantly, depending on the complexity of the surgery and the patient’s postoperative recovery. Morbidity rates can include infections, prolonged wound healing, and other surgery-related complications. Specific data on these outcomes were not clear or available in this SR; therefore, it is important to acknowledge these limitations and suggest areas for future research to better understand and optimize surgical treatment strategies.

This SR revealed that, in 81.3% of studies, surgical intervention was necessary as the primary choice for complication management or as a rescue operation. All included studies consistently described exploratory laparoscopy, which involved surgical treatment through various forms of fistula resection and suturing.

Given the variability in treatment approaches and the absence of a single best treatment strategy for this rare complication, it is essential to emphasize the fundamental surgical principles that should guide management. These principles include optimizing the patient’s nutritional status, resecting devitalized or infected tissue, ensuring effective drainage, and creating a tissue buffer between the affected organs. By adhering to these core principles, surgeons can tailor their approach to the specific clinical circumstances of each patient, potentially improving outcomes despite the challenges posed by the complexity of these studies.

### Algorithm-Based Approach

Among the reviewed studies, Tan et al. attempted to develop an algorithm-based approach for diagnosing and managing leaks, including one GCF following LSG. Their management strategy incorporated laparotomy, laparoscopy, endoscopic covered stenting, percutaneous radiologically guided drainage, jejunal enteric feeding, and total parenteral nutrition. While this approach could inform future studies, the study needed more statistical power to be considered robust. The proposed algorithm was based on a small cohort of only 14 patients treated with varying methods, and it remains to be seen how the single GCF was explicitly managed. Considering each procedure’s unique benefits and limitations, only larger, well-matched cohorts with reliable follow-up can provide definitive guidance for such management strategies [25].

Another systematic review by Bawa et al. on gastrocutaneous fistulas detailed the steps involved in the investigation and management strategy. All included studies initially attempted medical management, comprising antibiotics, skin protection, and artificial nutrition [26]. In this SR, an explicit treatment strategy was outlined. Specifically, 75.0% of the studies employed a nasojejunal tube for enteral nutrition to optimize the patient’s nutritional status, and 37.5% reported using antibiotics as a pre-treatment measure. Evidence from other SRs supports these approaches, indicating they are recommended practices [26]. However, it is essential to note that these interventions with antibiotics were not consistently presented across all studies in this review or other SR [38]. Therefore, implementing nasojejunal tube feeding for nutritional support and using antibiotics should be considered as primary actions in managing gastrocutaneous fistulas.

Furthermore, this SR could not build any form of algorithm, as 13 out of 16 studies were single-case reports. One cohort had 33 patients, with only one GCF case as a secondary complication, and another cohort was unable to provide conclusive results, as over 52.5% of patients who failed one or more double pigtail treatments after 12 months required laparoscopy as rescue surgery.

### Limitations

The primary limitations of this review include the variability in surgical techniques and postoperative management protocols and significant missing data regarding patient signs and symptoms, antibiotic treatment, hospital stay, and other clinical parameters. These are crucial for developing any predictive model and complicate the generalization of results to perform a meta-analysis.

Additionally, the predominance of single case reports and small sample sizes introduce a high risk of bias and confounding factors. The retrospective nature of most studies and the lack of comprehensive clinical data, such as patient signs, symptoms, and antibiotic treatments, hinder the development of reliable predictive models and treatment algorithms. Furthermore, we excluded marginal ulcers, which could have required a different treatment approach in various other MBS procedures. Larger, well-matched cohorts with reliable follow-up are necessary for more definitive conclusions.

## Conclusion

This systematic review concludes that surgical intervention should be the recommended approach for managing GCF following specific staple line leaks in LSG. The majority of GCF studies occurred more than six months post-surgery, indicating that it is a late complication. CT was the most frequently used diagnostic tool. Although over-the-scope clips and stents were employed, their efficacy as primary treatments remains questionable due to limited and inconsistent study outcomes.

## Supplementary Information

Below is the link to the electronic supplementary material.

Supplementary file1 (DOCX 31 KB)

## Data Availability

No datasets were generated or analysed during the current study.
